# Extensive Diversity and Prevalent Fluconazole Resistance among Environmental Yeasts from Tropical China

**DOI:** 10.3390/genes13030444

**Published:** 2022-02-28

**Authors:** Yiwei Liu, Zhongyao Chen, Jingyuan Li, Zhiqing Zhu, Sibei Pang, Jianping Xu, Jinyan Wu

**Affiliations:** 1 Public Laboratory, Hainan Medical University, Haikou 571199, China; karryon520921@163.com (Y.L.); y160301160@163.com (Z.C.); lijingyuan@personalbio.cn (J.L.); zzq13876456423@163.com (Z.Z.); jshmpangsibei@163.com (S.P.); 2 Key Laboratory of Tropical Translational Medicine of Ministry of Education, Hainan Medical University, Haikou 571199, China; 3 Department of Biology, McMaster University, Hamilton, ON L8S 4K1, Canada

**Keywords:** environment yeast, *Candida*, *Rhodotorula*, *Candida tropicalis*, fluconazole-resistance

## Abstract

Yeasts play important roles in both the environment and in human welfare. While some environmental yeasts positively contribute to nutrient cycling and food production, a significant number of yeast species are opportunistic human pathogens, including several that are tolerant/resistant to commonly used antifungal drugs. At present, most of our understanding of environmental yeasts has come from a few terrestrial environments in selected geographic regions. Relatively little is known about yeast diversity in tropical environments and their potential impacts on human health. Here, we characterize culturable yeasts in 968 environmental samples from eight regions in tropical China. Among the 516 soil, 273 freshwater, and 179 seawater samples, 71.5%, 85.7%, and 43.6% contained yeasts, respectively. A total of 984 yeast isolates were analyzed for their DNA barcode sequences and their susceptibilities to fluconazole. DNA sequence comparisons revealed that the 984 yeast isolates likely belonged to 144 species, including 106 known species and 38 putative novel species. About 38% of the 984 isolates belonged to known human pathogens and the most common species was *Candida tropicalis*, accounting for 21% (207/984) of all isolates. Further analyses based on multi-locus sequence typing revealed that some of these environmental *C. tropicalis* shared identical genotypes with clinical isolates previously reported from tropical China and elsewhere. Importantly, 374 of the 984 (38%) yeast isolates showed intermediate susceptibility or resistance to fluconazole. Our results suggest that these environmental yeasts could have significant negative impacts on human health.

## 1. Introduction

Yeasts are unicellular fungi with a typical vegetative growth by budding or fission. They are widely distributed in natural environments such as in oceans and seas, estuaries, rivers, lakes, and soils, and are associated with plants and animals [1,2,3,4]. So far, over 1500 yeast species belonging to 100 genera have been described. However, a much higher number of yeast species likely exists in the Earth’s biosphere. Some of these have been isolated as pure cultures but have not been formally described [1,2,3,5], and many remain to be cultured. Several yeast species play important roles in biofuel production and in the food industry such as for brewing, baking, vitamin production, and food preservation. These yeasts convert plant and animal organics to ethanol and other useful by-products. On the other hand, some yeasts are opportunistic pathogens to animals and humans. For example, a number of species in the genus *Candida* can invade many body sites and cause a range of infections, from skin and mucosal infections to systemic infections. Among the *Candida* species, several have been reported from a diversity of environments, including trees and soil. Furthermore, three of those species, *Candida auris*, *Candida glabrata*, and *Candida krusei* (syn. *Issatchenkia* *orientalis* and *Pichia kudriavzevii*), are known to be intrinsically resistant/tolerant to several common antifungal drugs, making their infections extremely difficult to treat [6,7,8,9,10,11]. Thus, understanding the distributions of yeasts in natural environments could have significant practical benefits, including for helping develop better strategies for managing yeast infections.

Aside from intrinsically drug-resistant yeast species in the environment that could impact human health, most yeasts are also capable of acquiring drug resistance traits. For example, our previous studies revealed prevalent drug-resistant yeast strains in tropical China for several species that are known to be typically susceptible to antifungal drugs in most other geographic populations [12,13]. Indeed, acquired drug resistance was found in strains colonizing the oral cavity of hosts who had never taking any antifungal drugs prior to sampling [12,13]. Because the human oral cavity is generally considered only a transient entry point for food and drinks into the gastrointestinal tract, and the oral mucosal surface is unlikely an effective site for selecting drug-resistant microorganisms, the presence of drug-resistant yeasts in oral cavities of hosts has been proposed as evidence of environmentally originated drug-resistant strains that subsequently colonized these hosts. Similar to these drug-resistant yeasts, triazole fungicides in agriculture have been suggested as key contributors to the recent emergence and rapid spread of multiple triazole-resistant strains of the filamentous human fungal pathogen *Aspergillus fumigatus* in many parts of the world, including China [14,15].

Due to its tropical climate, Hainan province is one of the most agriculturally productive regions in China. A large number of fruits, vegetables, and cereal crops are produced year-round in Hainan. In addition, Hainan produces a significant portion of seeds for several crops such as rice for most of China. Although the use of pesticides and fungicides in agriculture has been tightly controlled over the past decade, their overall use has remained at a relatively high level to ensure agricultural productivity and food security for the rest of the country [16]. These agricultural fungicides could have selected for drug-resistant yeasts in the environment that may transmit to humans via food and water. Furthermore, due to the tropical climate, Hainan is also a popular destination for tourists from both mainland China as well as outside of China in winter months. Therefore, it is important and necessary to understand the characteristics and potential health effects of environmental yeasts in ecological niches closely related to humans, such as farmlands, rivers, lakes, and beaches. The objective of this study was to investigate the diversity of yeasts and their fluconazole susceptibility profiles from natural environments, including soils, freshwaters, and seawaters in Hainan province in tropical China.

## 2. Materials and Methods

### 2.1. Sample Collection

**Figure 1:**
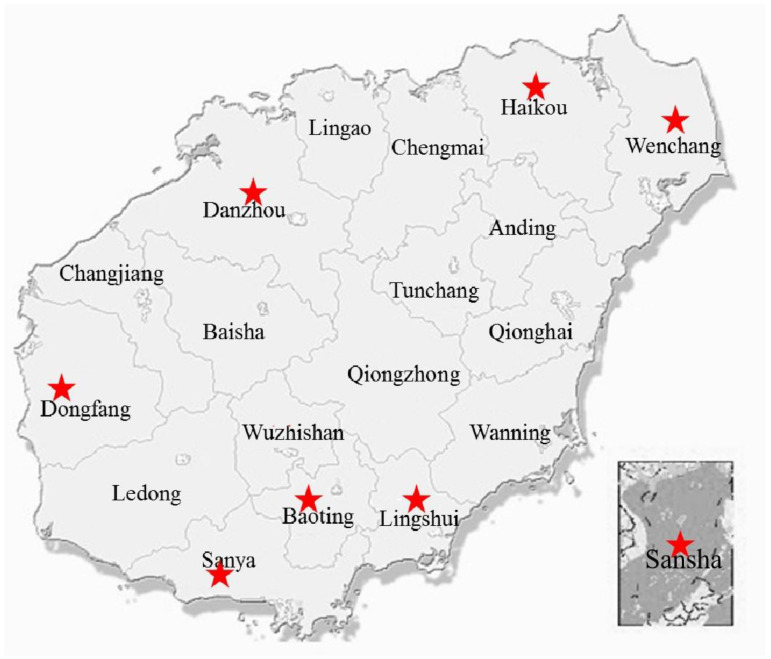
Geographic map of Hainan province in tropical China showing sites where environmental samples were collected for this study. The eight municipalities where samples were collected are marked by red stars.

We collected environmental samples from eight municipalities across Hainan province in tropical China during the summer of 2019 (Figure 1 and Table S1). All three types of samples (soil, freshwater, and seawater) were collected from the municipalities, except for Baoting, which is landlocked, so only soil and freshwater samples were collected. The soil samples were collected from orchards, vegetable gardens, and crop fields. Within each field, we collected 5–10 samples of topsoil (approximately 5 g each) between 1 and 3 cm from the surface, using a sterile spatula and gloves. The soil samples were at least 5 m from each other. Each soil sample was stored in a separate sterile plastic bag with a sealing strip. The freshwater samples were collected from rivers, lakes, ditches, and irrigation ducts. Within each water system, we collected 5–10 samples of surface water (2–3 mL each) at least 5 m from each other using sterile glass pipettes and stored the samples in 5 mL sterile plastic tubes. Seawater samples were collected from around beaches surrounding Hainan Island and Sansha Island. Around each beach, we collected 5–10 samples (2–3 mL each) of surface seawater at least 5 m from each other using sterile glass pipettes and stored them in 5 mL sterile plastic tubes. All collected samples were stored in a cooler with ice and transported to the laboratory on the same day. In total, we investigated 968 environment samples, including 516 soil samples, 273 freshwater samples, and 179 seawater samples (Table S1). These samples were distributed among the following municipalities: Haikou (63 soil, 28 freshwater, 29 seawater), Wenchang (67 soil, 37 freshwater, 27 seawater), Lingshui (71 soil, 24 freshwater, 24 seawater), Baoting (68 soil, 67 freshwater), Danzhou (81 soil, 44 freshwater, 28 seawater), Dongfang (61 soil, 34 freshwater, 32 seawater), Sanya (68 soil, 32 freshwater, 33 seawater), and Sansha (37 soil, 7 freshwater, 6 seawater) (Table S1).

### 2.2. Yeast Isolation

For each soil sample, approximately 0.1 g was added into 1 mL sterile YEPD broth composed of 2% (weight/volume) yeast extract, 1% peptone, 2% dextrose, and the antibiotic chloramphenicol (50 µg/mL) in 5 mL culture tubes and incubated in a roller drum at 35 °C for 24 h. The 35 °C temperature was chosen to mimic the average outdoor temperature at noon in the summer in Hainan. For each freshwater and seawater sample, 1 mL of the collected sample was aliquoted into a fresh 5 mL culture tube and centrifuged at 12,000× *g* rpm for 1 min to collect the microbial cells and sediment. After the supernatant was discarded, 1 mL of YEPD broth was added to the culture tubes and incubated in a roller drum at 35 °C for 24 h to enrich yeast population. The supernatants were streaked onto Sabouraud dextrose agar (SDA) medium for isolation of yeasts, one SDA plate for each tube. After incubation at 35 °C for 48 h, morphologically yeast-like colonies were randomly selected and streaked onto fresh YEPD plates to obtain single colonies. If more than one morphology type of yeast was present on one plate, one representative colony of each type was separately streaked onto a new plate for single colonies. After 2–3 days of incubation, we randomly picked one single colony per yeast isolate from each plate for storage and subsequent analyses. All purified yeasts isolated from these samples were stored in Sabouraud dextrose broth containing 30% glycerol in a −80 °C freezer.

### 2.3. Identification of Yeast Species

Actively growing yeast cells from the purified cultures were harvested and their genomic DNA extracted following a yeast miniprep protocol described previously [17]. To identify the species affiliation of these yeast isolates, we used the universal fungal DNA barcode primers ITS1 and ITS4 to amplify the inter transcribed spacer (ITS) regions of the nuclear ribosomal RNA gene cluster through polymerase chain reaction (PCR), following protocols described previously [13]. The amplified ITS PCR products were sequenced at BGI Shenzhen, China. Yeast ITS sequences were compared to those in the GenBank through the BLASTN search option. We assigned species identities to our ITS sequences at a sequence similarity threshold of 98.41% to existing sequences in databases, as described by Samarasinghe et al. [18]. This threshold was previously determined to reflect the nucleotide difference between sister yeast species at the ITS locus based on an analysis of 9000 fungal sequences [19]. Yeasts with ITS sequences below this threshold to any known yeast species were considered putative novel species.

### 2.4. Fluconazole Susceptibility Testing

For all yeast isolates obtained from our soil, freshwater, and seawater samples, we determined their susceptibility to fluconazole, the most commonly used antifungal drug in China for treating patients with yeast infections. We used the agar disk diffusion method to determine their susceptibility, following the CLSI M44-A2 guidelines [20]. The fluconazole disks were all obtained from ROSCO [21]. We used two reference strains as recommended by CLSI M44-A2 [20] and followed the interpretive criteria of ROSCO [21] for susceptible (S), intermediate (I), and resistance (R) for this drug. The same procedure for determining fluconazole susceptibility of yeasts has been described in previous studies [13].

### 2.5. Multi-Locus Sequence Analyses of C. tropicalis

Among the yeast species in tropical China, the population genetics of one species, *Candida tropicalis*, have been examined using multi-locus sequence typing (MLST) based on DNA sequence variation at six loci. All previously analyzed strains of *C. tropicalis* from tropical China were from human hosts. In the current study, we are interested in the relationships between strains of *C. tropicalis* from the environments and those from humans reported previously from tropical China, and to a lesser extent, from other geographic locations. To analyze their relationships, a subset of the *C. tropicalis* isolates obtained in this study were genotyped by MLST at the same six loci (*ICL1*, *MDR1*, *SAPT2*, *SAPT4*, *XYR1*, and *ZWFa1*) used previously. The MLST analysis followed protocols described previously [22]. To ensure that all heterozygous sites were accounted for, all sequence chromatograms were manually inspected. These sequences were then compared with the existing sequences in the *C. tropicalis* MLST database (http://pubmlst.org/ctropicalis/, accessed on 15 January 2022 to obtain a sequence profile for each locus of each strain and a combined diploid sequence type (DST) for each strain based on the sequence profiles at all six gene fragments [22]. The new sequence profiles at each individual locus and at the combined six loci that were absent in the original database were assigned new sequence and DST numbers (Table S2, highlighted in red). The sequences for all our sequenced strains at the six loci have been deposited in the *C. tropicalis* MLST database. To analyze the relationships among sequences from environment samples in this study and those from human hosts from tropical China, as reported in our previous study [22], we aligned the sequences in the same FASTA file. The sequence relationships at each locus and strain relationships based on sequences at the combined 6 loci were determined through cluster analysis using UPGMA (unweighted pair group method using their arithmetic averages) of the MEGA software [23]. Each polymorphic nucleotide site was treated as an informative site and alternative nucleotides at each locus as different alleles. To infer the patterns of genetic variation, the sequences were imported into the computer program GenAlEx 6.5 [24]. The population genetic parameters, such as the number of polymorphic nucleotide sites within each gene fragment and allelic diversity in each ecological population, were estimated. The analysis of molecular variance (AMOVA) was performed to estimate the relative contributions of ecological separation to the overall genetic variation in *C. tropicalis*.

### 2.6. Statistical Analyses

We quantified the diversity of yeast populations at our sampling sites by calculating the Shannon diversity index using the package Vegan v.2.5–7 [25] (pp. 5–7). All statistical comparisons among samples were conducted in GraphPad Prism 8 using the chi-square test. Statistical significance was defined as a p-value of less than 0.05. Sequence comparisons and population genetic analyses were performed as described above.

## 3. Results

### 3.1. Yeast Isolation and Species Identification

**Figure 2:**
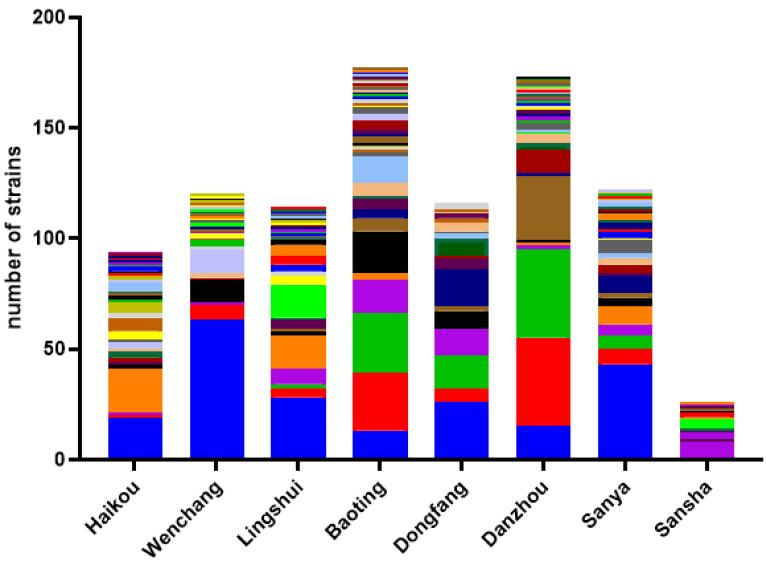
Diversity and abundance of culturable environmental yeasts within each of the eight geographic samples. Labels on the X-axis represent the eight geographic regions. The yeast diversity of each geographic region is represented by a stacked bar plot. In the stacked bars, each color represents a unique species, and the height of the colored sections indicates the abundance of that species in the population. For detailed prevalence of each species at each region, see Table S1.

In this study, we successfully isolated yeasts from 681 out of 968 environmental samples. Among the three types of samples, freshwater samples had the highest yeast isolation rate at 85.7% (234/273), followed by soil (369/516, 71.5%) and seawater (78/179, 43.6%) (Table 1). Aside from the freshwater and seawater samples from Sansha municipality (the southernmost site) that did not yield any yeast, yeasts were isolated from all types of the remaining environmental samples from each of the eight municipalities (Table 1 and Table S1). However, differences in yeast isolation rate were observed among geographic regions and ecological niches (Figure 2; Table 1 and Table S1; *p* < 0.0001). For example, the yeast isolation rates among the eight municipalities varied from 38% in Sansha to 85.93% in Baoting. Among the three ecological types, the yeast isolate rates showed significant differences between soil and seawater as well as between freshwater and seawater (*p* < 0.0001 in all comparisons). However, the differences between soil and freshwater samples were statistically insignificant (*p* = 0.259). We note that the differences in yeast isolation rates were not related to the sample sizes (*p* = 0.468).

From the 681 environmental samples that contained yeasts, many samples had more than one type of yeast colony. Consequently, we obtained more yeast isolates (984) than the number of environmental samples (681) containing yeasts. Based on their ITS sequences, the putative taxonomic associations were assigned to all 984 isolates using the 98.41% sequence identity cut-off to homologous ITS sequences in the NCBI and UNITE databases. These strains were categorized into 106 known species belonging to 40 genera and 38 putative new species. The most common genus was *Candida*. Indeed, there were 24 species in the broad paraphyletic genus *Candida*. Specifically, seven *Candida* species belonged to the *Lodderomyces* clade; three belonged to the *Pichia* clade; two belonged to the *Nakaseomyces* clade; and clades *Clavispora*, *Candida glaebosa*, *Ogataea*, and *Yamadazyma* were represented by one species each. The eight remaining species in genus *Candida* were not assigned to a known clade (Table S3). Overall, the 106 species belonged to two phyla (Ascomycota and Basidiomycota), six Classes, ten Orders, and 17 Families. However, it should be noted that two genera, *Candida* and *Diutina*, are both paraphyletic and do not currently have defined Family associations (incertae sedis).

In each of the eight municipalities, a diversity of yeast species was found (Figure 2; Table 1 and Table S1). Overall, the most common species was *C. tropicalis* (207/984, 21%), followed by *C. krusei* and *Torulaspora* *delbrueckii* (90/984, at 9.1% each), and 82 of the 144 species/putative species were represented by one strain each. Similar to the significant differences in yeast isolation rates among geographic regions, yeast species distributions were also significantly different among the eight regions (*p* < 0.0001). For example, Wenchang was dominated by *C. tropicalis* (Figure 2). Among the three ecological niches, the diversity of culturable yeasts from soil was significantly higher than those from freshwater and seawater (*p* < 0.003 in both comparisons). However, although there was a higher number of putative novel species in the soil samples (22) than freshwater (14) and seawater (2) samples, the percentages of species being novel among the ecological niches were statistically insignificant (*p* = 0.553). Instead, the differences were likely due to the different sample sizes that we analyzed here among the three ecological niches. Geographically, the putative novel species were also broadly distributed among six of the eight municipalities. Only samples from Lingshui, southeast Hainan Island, and from Sansha Island failed to yield potential novel species.

### 3.2. Diversity and Abundance of Culturable Environment Yeast Populations from Each Geographic Region

As described above, the abundance and diversity of yeasts in soil, freshwater, and seawater samples varied significantly among the eight geographic regions. Below, we briefly describe yeast diversity in each of the eight geographic regions. The order of description below is based on the alphabetic order of the first letter of the names of the eight municipalities.

#### 3.2.1. Haikou

Haikou is located in the northern part of Hainan Island and is the capital city of Hainan province. The yeast isolation rates of soil, freshwater, and seawater samples from Haikou were 69.8%, 71.4%, and 20.7%, respectively. The culturable soil yeast population in Haikou consisted of similar numbers of ascomycete and basidiomycete species (13 and nine, respectively). Two yeast species each in Ascomycota and Basidiomycota were found in seawater samples. From the freshwater samples, we isolated 12 ascomycete species and six basidiomycete species. Overall, the culturable yeasts in soil samples were dominated by *C. pseudolambica* (15/62, 24.2%) and *C. tropicalis* (14/62, 22.6%). The culturable freshwater yeasts were dominated by three species: *C. tropicalis* (5/29, 17.2%), *C. pseudolambica* (4/29, 13.8%), and *Diutina* *mesorugosa* (3/29, 10.3%). The other 15 species only had one representative strain each. The four yeast species isolated from seawater samples in Haikou were *C. parapsilosis* (two strains), *Cutaneotrichosporon* *dermatis* (one strain), *D.* *mesorugosa* (two strains), and *Rhodotorula* *mucilaginosa* (one strain). A total of three potential novel species were found in Haikou, one from soil and two from freshwater (Table 1 and Table S1).

#### 3.2.2. Wenchang

Wenchang is located in northeastern part of Hainan Island. The yeast isolation rates of soil, freshwater, and seawater samples from Wenchang were 73.1%, 75.7%, and 59.3%, respectively. The culturable yeast populations in Wenchang soil and freshwater samples were dominated by ascomycetes (16 species in soil, seven in freshwater). The remaining eight species in soil and one in freshwater were basidiomycetes. The culturable seawater yeast population in Wenchang consisted of three ascomycete species and two basidiomycete species. *C. tropicalis* was the most common species in all three ecological niches in Wenchang: in soil (25/66, 37.9%), freshwater (26/41, 63.4%), and seawater (12/17, 70.6%). Four potential novel species were found in Wenchang, two from soil and two from seawater (Table 1 and Table S1).

#### 3.2.3. Lingshui

Lingshui is located on the southeastern side of Hainan Island. The yeast isolation rates of soil, freshwater, and seawater samples from Lingshui were 78.9%, 95.8%, and 37.5%, respectively. The culturable yeast populations in Lingshui soil and freshwater samples were dominated by ascomycetes (16 species in soil, 11 in freshwater). The remaining five species in soil samples and three species in freshwater samples belonged to basidiomycetes. The culturable seawater yeast population in Lingshui consisted of similar numbers of ascomycetes and basidiomycetes, at three and four, respectively. The culturable soil yeast population was dominated by *C. tropicalis* (24/72, 33.3%), *C. intermedia* (10/72, 13.9%), and *C. pseudolambica* (9/72, 12.5%). The most prevalent yeast species in freshwater samples were *C. pseudolambica* (6/31, 19.4%), *C. intermedia* (5/31, 16.1%), *Meyerozyma* *caribbica* (5/31, 16.1%), *C. tropicalis* (3/31, 9.7%), and *C. guilliermondii* (3/31, 9.7%). In seawater samples from Lingshui, *C. parapsilosis* (4/11, 36.4%) was the most common species. Interestingly, all yeast isolates from Lingshui could be assigned to known species (Table 1 and Table S1).

#### 3.2.4. Baoting

Baoting is located in the south center of Hainan Island and does not border with the sea. Thus, only two ecological samples, soil and freshwater, were analyzed. The yeast isolation rates of soil and freshwater samples from Baoting were 88.2% and 83.6%, respectively. The culturable yeasts in the soil and freshwater samples were dominated by ascomycetes (22 species in soil, and 18 species in freshwater). The remaining three species in the soil samples and eight species in freshwater samples were basidiomycetes. At the species level, the five most common culturable soil yeasts were *T.* *delbrueckii* (24/101, 23.8%), *C. krusei* (17/101, 16.8%), *M.* *caribbica* (10/101, 9.9%), *C. quercitrusa* (10/101, 9.9%), and *C. tropicalis* (6/101, 5.9%). Two species (*Trichosporon* *asahii* and *Wickerhamomyces* *sydowiorum*) were represented by four strains each in the soil samples (Table S1). Four (*C. krusei*, *C. tropicalis*, *T. asahii*, and *M. caribbica*) of these top seven species from the soil samples were shared with top species from the freshwater samples. The following six yeast species were the most frequent in the freshwater samples: *T.* *asahii* (15/90, 16.7%); *C. krusei* (8/90, 8.9%); *C. tropicalis* (7/90, 7.8%); and *Diutina rugosa*, *Kodamaea* *ohmeri*, and *M. caribbica* with five strains each (5/90, 5.6%). Overall, among samples from the eight geographic regions, those from Baoting contained the highest number of putative new species (four from soil and 11 from freshwater) (Table 1 and Table S1).

#### 3.2.5. Dongfang

Dongfang is located on the western side of Hainan Island. The yeast isolation rates of soil, freshwater, and seawater samples from Dongfang were 88.5%, 97.1%, and 25%, respectively. Ascomycota was the dominant phylum in Dongfang, including 12 species from soil, seven from freshwater, and three from seawater samples. The remaining species (six from soil, one from freshwater, and two from seawater) belonged to Basidiomycota. The culturable soil yeasts were dominated by *Rhodotorula* *toruloides* (12/69, 17.4%), *W.* *sydowiorum* (10/69, 14.5%), *M.* *caribbica* (8/69, 11.6%), and *Rhodotorula sp.* New (8/69, 11.6%). The most common culturable freshwater yeast species was *C. tropicalis* (22/48, 45.8%). A total of five species were isolated from seawater samples in Dongfang: *T.* *delbrueckii* (5/11, 45.5%), *T.* *asahii* and *W.* *sydowiorum* (two strains each), and *M.* *caribbica* and *Trichosporon asteroids* (one strain each). There were eight potential new species in the Dongfang sample, all of which were from the soil (Table 1 and Table S1).

#### 3.2.6. Danzhou

Danzhou is located on the northwestern side of Hainan Island. The yeast isolation rates of soil, freshwater, and seawater samples from Danzhou were 50.6%, 97.7%, and 100%, respectively. The culturable yeast populations in Danzhou soil, freshwater, and seawater samples were dominated by ascomycetes (20 species in soil, 10 in freshwater, and 6 in seawater). The remaining nine species in soil and one species in seawater were basidiomycetes. Except for *Dirkmeia* *churashimaensis*, which had 10 strains, the other culturable soil yeast species were all relatively uniformly distributed, with each of the remaining 28 species represented by 1–3 strains each. Different from the culturable soil yeast population, the freshwater and seawater yeasts belonged to relatively fewer species with skewed frequencies. Specifically, the culturable freshwater yeasts were dominated by *T.* *delbrueckii* (23/76, 30.3%), *D. rugosa* (20/76, 26.3%), *C. krusei* (18/76, 23.7%), and *C. tropicalis* (9/76, 11.8%). Interesting, the same four yeast species dominated the culturable seawater yeast population but with different orders of prevalence and frequencies: *C. krusei* (18/49, 36.7%), *T. delbrueckii* (16/49, 32.7%), *D. rugosa* (7/49, 14.3%), and *C. tropicalis* (4/49, 8.2%). All six potential novel species in Danzhou came from soil samples (Table 1 and Table S1).

#### 3.2.7. Sanya

Sanya is the located on the southern part of Hainan Island. The yeast isolation rates of soil, freshwater, and seawater samples from Sanya were 67.7%, 96.9%, and 33.3%, respectively. The culturable yeast populations in Sanya soil, freshwater, and seawater samples were all dominated by ascomycetes (17 species in soil, 10 species in freshwater, and all 7 species in seawater samples). The remaining four species in soil and six species in freshwater were basidiomycetes. *C. tropicalis* was the most common species in soil (26/61, 42.6%) and freshwater (17/46, 36.9%). The most common species in seawater were *T.* *delbrueckii* and *W.* *sydowiorum* (both of 6/17, 35.3%). Two potential novel species were found in Sanya, one from soil and one from freshwater (Table 1 and Table S1).

#### 3.2.8. Sansha

Sansha Island is the southernmost sampling site in this study and is distantly separated from other seven sites. No yeast was isolated from freshwater or seawater samples from Sansha. The soil yeast isolation rate was 51.4%. The culturable soil yeast population in Sansha was dominated by ascomycetes (11 species) and the remaining two yeast species belonged to basidiomycetes. The top three most prevalent species in Sansha soil samples were *M.* *caribbica* (9/26, 34.6%), *Kazachstania aquatic* (4/26, 15.4%), and *Torulaspora* *indica* (3/26, 11.5%). No potential novel species were found in Sansha (Table 1 and Table S1).

### 3.3. Putative Novel Yeast Species

**Figure 3:**
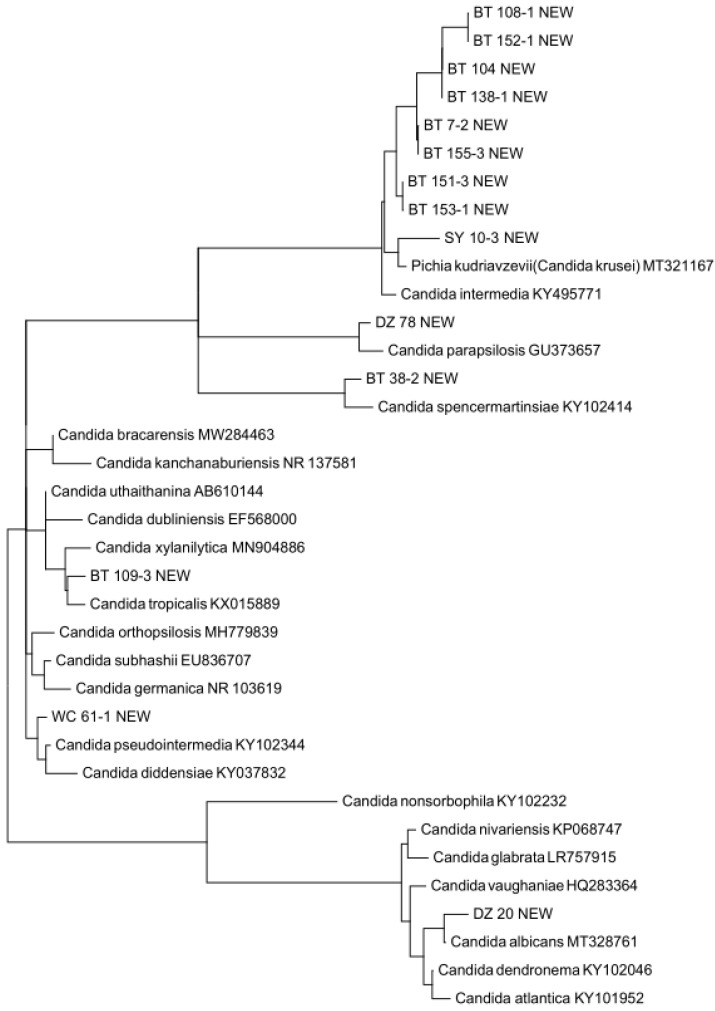
Maximum likelihood tree showing the relationships between ten putative novel *Candida* species and their closely related known species based on rDNA ITS sequences. The geographic origins of these putative novel species are indicated as two-letter abbreviations, where BT = Baoting, DZ = Danzhou, and WC = Wenchang. The tree was constructed using RaxML with 1000 bootstraps.

**Figure 4:**
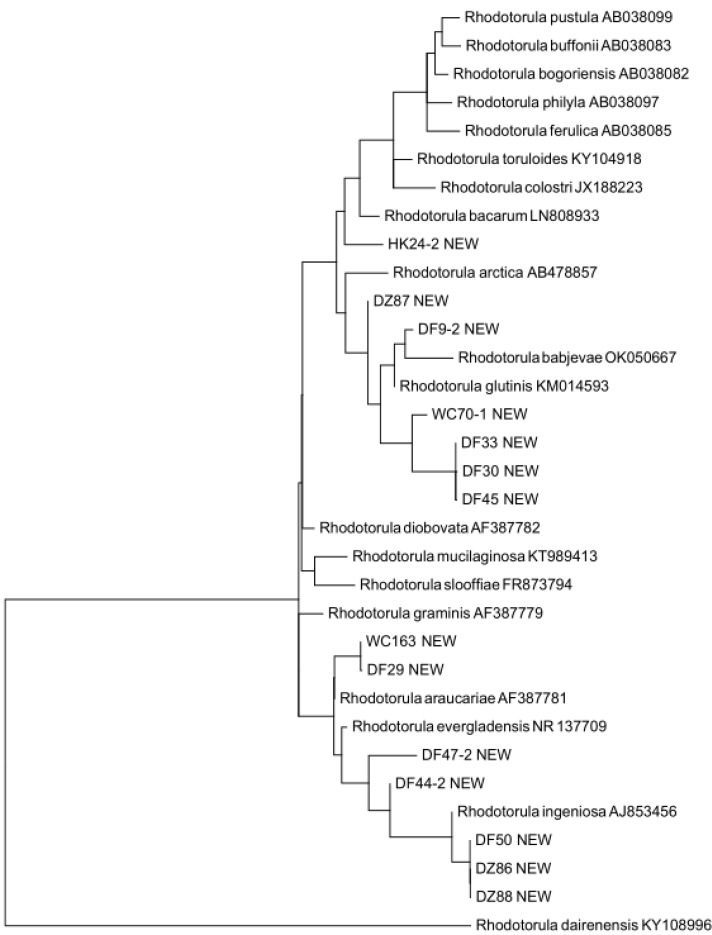
Maximum likelihood tree showing the relationships between nine putative novel *Rhodotorula* species and their closely related known species based on rDNA ITS sequences. The geographic origins of these putative novel species in our sample are indicated as two-letter abbreviations, where HK = Haikou, DZ = Danzhou, WC = Wenchang, and DF = Dongfang. The tree was constructed using RaxML with 1000 bootstraps.

Our yeast population included 38 potentially novel species from six municipalities. Samples from Lingshui and Sansha municipalities did not yield any putative novel yeast species. Among these 38 putative novel species, 22 were found in soil samples, 14 in freshwater samples, and 2 in seawater samples (Table S1). We determined the most closely related genera for all 38 putative species by running BLASTN searches in the UNITE database. Our comparisons revealed that these 38 putative novel species could be categorized into 11 genera (six in ascomycetes, five in basidiomycetes) with *Candida* and *Rhodotorula* containing 14 and 15 potentially novel species, respectively (Table S4). For each genus, we constructed a maximum likelihood (ML) tree using RaxML with 1000 bootstraps [26] to determine the potential taxonomic placements of the novel species with respect to other closely related known species of that genus. Our ML trees confirmed the separation of newly discovered putative species from their closely related known species. Figure 3 and Figure 4 show the distinctness of the 14 potentially novel *Candida* species and 15 putative *Rhodotorula* species we isolated in this study. We note that because gaps (indels) were excluded in the ML phylogenetic analyses, some of the putative novel species showed very close relationships to each other in Figure 3 and Figure 4. However, none of the putative new species shown here had identical sequences to each other.

### 3.4. Pathogenic Yeast Species

**Figure 5:**
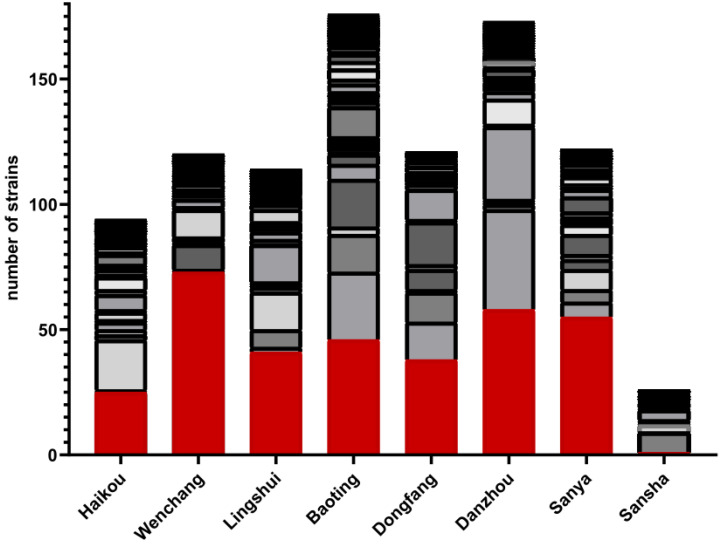
Prevalence of opportunistic yeast pathogens in environmental samples from eight municipalities in Hainan province in tropical China. Highlighted in red are the number of strains of pathogenic yeast species, and those in various shades of gray are not known to be pathogenic to humans.

Based on recent information on yeast trophism [2,25,27], the following 12 species are considered common opportunistic yeast pathogens of humans worldwide: *C. albicans*, *Candida dubliniensis*, *C. tropicalis*, *C. glabrata*, *C. guilliermondii* (syn: *Meyerozyma* *guilliermondii*), *C. krusei* (*Pichia kudriavzevii*), *C. lusitaniae* (syn: *Clavispora* *lusitaniae*), *C. parapsilosis*, *C. orthopsilosis*, *C. metapsilosis*, *Cryptococcus neoformans*, and *Cryptococcus deneoformans*. Among the 984 yeast isolates from our samples, 343 strains belonged to nine of these species, accounting for 38.4% of our environmental yeast population in Hainan (Figure 5). *C. tropicalis* was the most abundant and most widespread with 207 isolates originating from all three ecological niches and in the seven of the eight municipalities except Sansha. The remaining eight opportunistic human pathogens were *C. krusei* (90 isolates); *C. guilliermondii* (18 isolates); *C. parapsilosis* (9 isolates); *C. albicans* (5 isolates); *C. dubliniensis* (4 isolates); and *C. orthopsilosis*, *C. glabrata*, and *C. lusitaniae* (3 isolates each).

### 3.5. Profile of Fluconazole Susceptibilities

The results of our antifungal susceptibility testing of all the environmental yeasts are summarized in Table 2. The frequencies of isolates with intermediate (I), susceptible (S), and resistant (R) phenotypes to fluconazole varied widely among the yeast species. Overall, 56 known species and six putative new species had at least one isolate with the I and/or the R phenotypes to fluconazole (Table 2). The remaining yeast species, including four human opportunistic pathogens *C. parapsilosis* (nine isolates), *C. glabrata* (three isolates), *C. orthopsilosis* (three isolates), and *C. lusitaniae* (three isolates), representing 102 isolates total, had the S phenotype to fluconazole. However, the remaining five human opportunistic yeast pathogens showed various frequencies of resistance to fluconazole: (i) 25.4% of *C. tropicalis* isolates including eight intermediate and 43 resistant to fluconazole; (ii) 65.9% of *C. krusei* isolates including 33 intermediate and 23 resistant to fluconazole; (iii) 33.3% of *C. guilliermondii* isolates including two intermediate and four resistant to fluconazole; and (iv) one *C. albicans* isolate (20%) and two *C. dubliniensis* (40%) isolates resistant to fluconazole. Overall, 38% (374/984) of all yeast strains from our environmental samples were either intermediate or resistant to fluconazole.

### 3.6. Multi-Locus Sequence Typing of C. tropicalis in the Environment

Among the 207 isolates of *C. tropicalis*, 44 were selected for multi-locus sequence typing. These 44 isolates were from two of the eight geographic regions where clinical samples were analyzed previously [22]. We successfully obtained DNA sequences from all six loci (*ICL1*, *MDR1*, *SAPT2*, *SAPT4*, *XYR1*, and *ZWFa1*) for all 44 isolates (Table S2). The concatenated six gene sequences contained 2677 bp, and a total of 57 (2.51%) polymorphic nucleotide sites were found among these 44 strains (Table 3). Among the combined total of 78 locus-specific sequence types at the six gene fragments, 13 were new to the *C. tropicalis* MLST database and had never been reported from other geographic regions (Table S2, highlighted in red). The combined analyses of sequence information from the six gene fragments identified a total of 33 diploid sequence types (DSTs) among the 44 isolates (Table S2). Among these 33 DSTs, 10 have been reported previously and the remaining 23 DSTs were new to the database (Table S2, highlighted in red). The details about the genetic variation at each of the six gene fragments in the environmental population of *C. tropicalis* are described in Table 3. For comparison, similar information from the clinical population of *C. tropicalis* from Hainan is also presented in Table 3. At the individual locus level, compared to *C. tropicalis* from the clinical samples in Hainan [22], the most frequent *ICL1* genotypes in the clinical and environmental samples of *C. tropicalis* were the same (*ICL1* genotype 1). However, at other five loci, the most frequent genotypes were different between the clinical and environmental populations of *C. tropicalis* (Table S5).

Our previous study revealed that geographic separation among regional populations of *C. tropicalis* contributed less than 2% of the total genetic variation in the clinical populations of this yeast from Hainan province. In contrast, most genetic variations were found within individual strains (75%) and among strains within individual geographic regions (23%). Here, we compared the population genetic differences between the environmental and clinical *C. tropicalis* samples from Hainan reported previously. AMOVA analyses revealed that the ecological separation (clinical vs. environmental) contributed 6% to the total observed genetic variation and that this contribution was statistically significant (*p*-values = 0.001) (Table 4).

**Figure 6:**
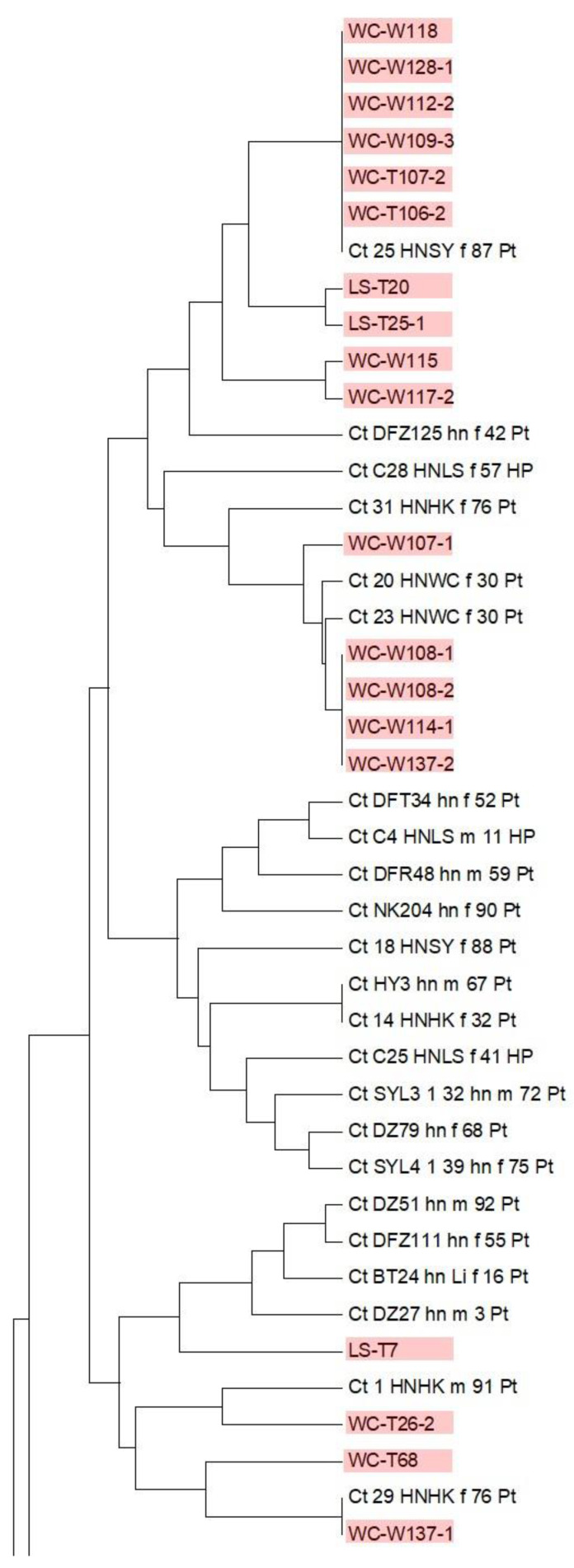

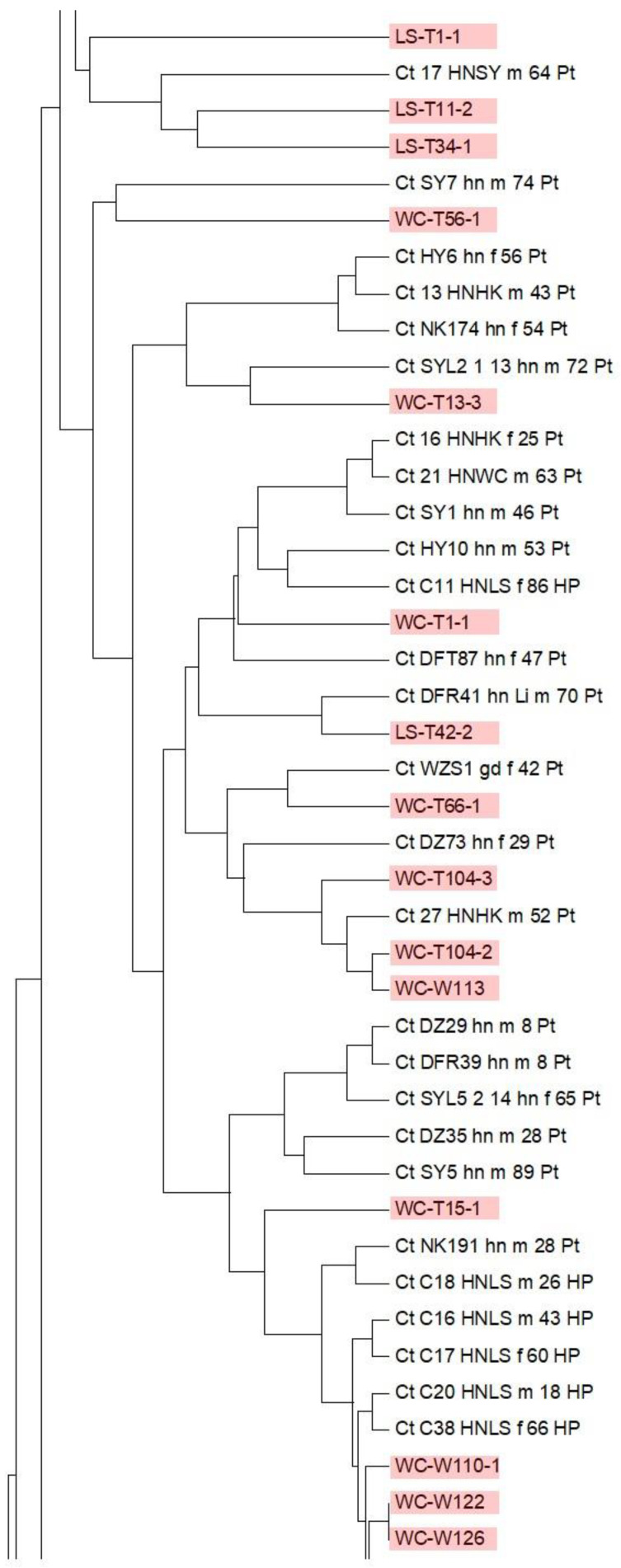

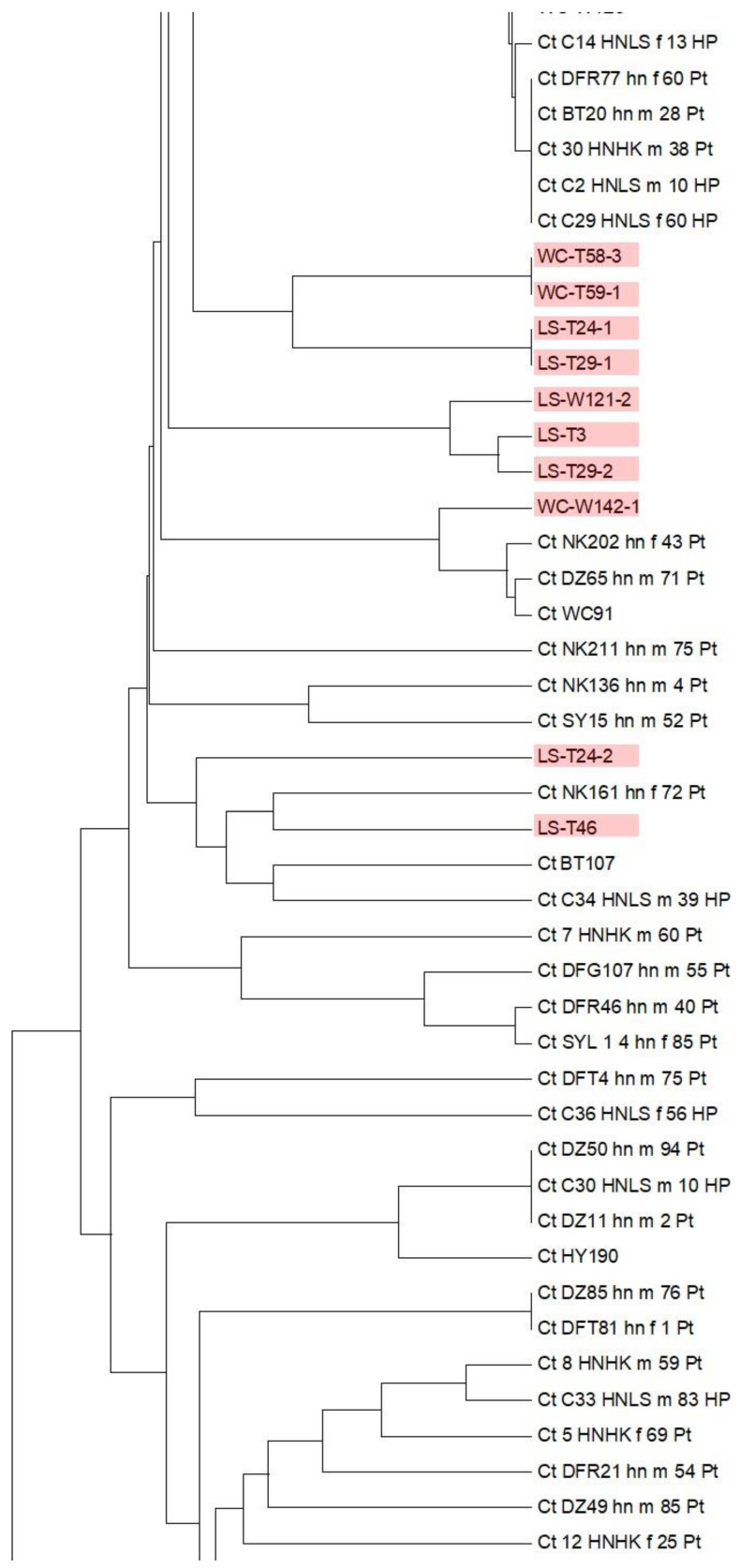

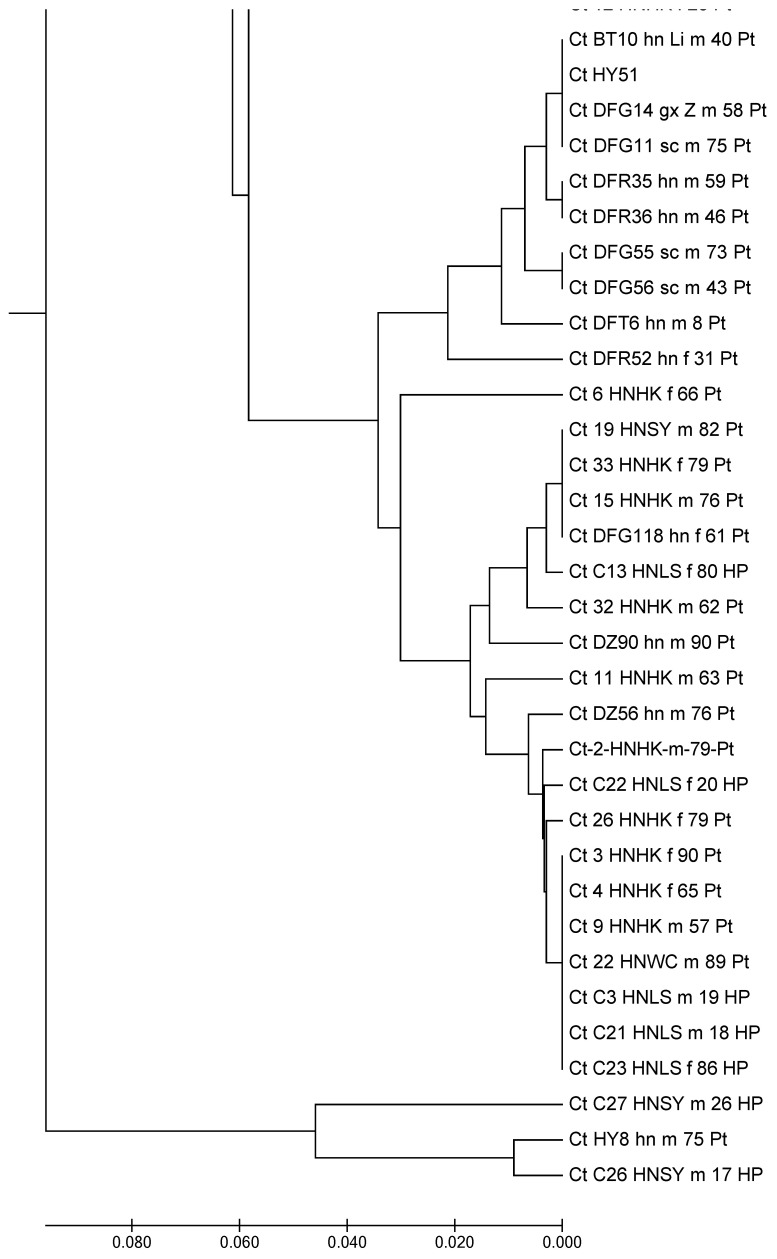
Relationships among strains of *C. tropicalis* from environmental and clinical sources in Hainan province in tropical China. Strains highlighted in red were from natural environments isolated in this study. The non-highlighted ones were from oral cavities of patients in Hainan reported in our previous study. For detailed strain information, see Ref. [22] and Table S4.

Among the 33 DSTs identified in the 44 environmental *C. tropicalis* strains, 10 (DST 99, 139, 436, 474, 522, 667, 894, 895, 897, and 959) were shared with those already in the *C. tropicalis* MLST database reported from the clinical population in Hainan province and/or from other geographic regions outside of Hainan. Specifically, DST 99 was shared with one strain from Beijing (China) and one strain from Colombia (South America); DST 139 was shared with several strains from Taiwan Island and one strain from Shenzhen (China); DST 436 and 474 were shared with those from clinical strains within Hainan (China); DST 522 was shared with several strains from Shanghai and one from Shenzhen (China); DST 667 and 959 were shared with several from Taiwan Island; and DST894, 895, and 897 were shared with those from Thailand. Several DSTs were shared among strains from different ecological niches in Hainan environments (Table 4 and Figure 6). The genetic relationships among the 160 isolates (44 environmental and 116 clinical) from Hainan based on concatenated sequences at all six loci are shown in Figure 6. None of the 44 environmental strains genotyped using MLST had an identical MLST genotype as clinical drug-resistant strains reported previously from Hainan.

## 4. Discussion

In this study, we surveyed yeasts across broad geographic regions and ecological niches in Hainan province in tropical China. We found that yeasts are prevalent in soil, freshwater, and seawater ecosystems in tropical regions in China. In addition, the yeast communities within each of the three ecological niches and of the eight sampled geographic areas were very diverse. Our DNA barcode sequence analyses indicated that our environmental yeasts likely contained 38 new species not reported previously in the literature. In addition, multi-locus sequence analyses of the most common yeast species *C. tropicalis* revealed close relationships between environmental strains and clinical strains infecting human hosts. Unfortunately, about 38% of the total 984 yeast isolates showed intermediate or resistance phenotypes to fluconazole, the most common antifungal drug for treating human yeast infections. Together, our results suggest both significant potentials and emerging threats of environmental yeasts from tropical China to humans.

Yeasts have been isolated from many natural and anthropogenic environments, including soil, freshwater, and marine environments [2,3,4]. They play important roles in the health of soils in agricultural, horticultural, grassland, and forest ecosystems [28]. However, most such studies have focused on samples from subtropical and temperate regions and relatively little is known about environmental yeast diversity in tropical regions, including in tropical China. Our study here thus complements earlier studies and provides baseline information about yeast diversity in this geographic region. For example, the potential novel species identified here establish a foundation for yeast taxonomy studies in tropical China. Similarly, the strains obtained in this study can help identify potential novel traits for industrial applications. In addition, the prevalence of fluconazole resistance among yeasts from across the eight geographic regions and ecological niches suggests the needs for a coordinated approach to identify the origins and spread of antifungal resistance across environments. Indeed, due to its tropical climate, Hainan province has been one of the agriculturally most intensive regions in China and a popular destination for tourists from both mainland China as well as outside of China. The identifications of genotypically identical and very similar strains of *C. tropicalis* between Hainan province and those outside of Hainan are consistent with the long-distance dispersal of yeasts in tropical regions and between tropical, subtropical, and temperate regions. Therefore, understanding the environmental yeast diversity in Hainan will have broad implications beyond Hainan province.

The most invasive yeast infections are frequently caused by pathogens from the genera *Candida* and *Cryptococcus* [29,30]. In our survey, we found a large number of strains belonging to opportunistic human pathogen species, with most of these in the genus *Candida*, including *C. tropicalis* and *C. parapsilosis*. However, no strain of the human pathogenic *C. neoformans* and *C. gattii* species complexes were found in any of the environmental samples from Hainan province. In a recent report by Samarasinghe et al. [2], no isolate belonging to any common yeast pathogens were isolated in their soil samples from several mountains in China. In contrast, both *C. tropicalis* and *C. parapsilosis*, as well as *C. catenulata* and *T. asahii* found in the Hainan environmental samples have been found in the marine waters around the tropical Andaman Islands in the Bengal Bay of the Indian Ocean [11]. However, different from the report by Arora et al. [11], where *C. auris* was reported in their samples, no *C. auris* was found in our survey from tropical China, including the seawaters. The results suggest that the two tropical marine waters, one in the South China Sea and the other in the Indian Ocean, likely have both shared and distinct yeast species and communities.

In this study, a total of 984 isolates were obtained and analyzed from 681 environmental samples from eight municipalities in Hainan province. Our analyses revealed that samples from these eight municipalities differed in their yeast isolation rates. For example, no yeast was isolated in the freshwater and seawater samples from Sansha. In addition, the yeast isolation rate and species diversity in Sansha soil samples were significantly lower than those in seven other municipalities. Moreover, all isolates from Sansha were susceptible to fluconazole (Table S6). The uniqueness of the Sansha yeast community was likely related to its unique geographic location (being the farthest away from other sites) and limited number of human residents. Such a result suggests that humans and human activities could have a significant influence on environmental yeasts, consistent with those reported by Samarasinghe et al. [2].

Aside from the common opportunistic yeast pathogens in the genus *Candida*, we also found several clinically rare but relevant yeast species in our samples, including *T. asahii* and *K. ohmeri*. *T. asahii* can cause invasive trichosporonosis in patients with hematological malignancies and other medical conditions associated with immunocompromised people [31,32,33]. *K. ohmeri* has been recognized as an emerging yeast pathogen that can cause a diversity of infections in both immunocompromised and some apparently immunocompetent humans, including fungemia, funguria, endocarditis, peritonitis, and wound infections [34,35,36,37]. Similarly, *Rhodotorula* species such as *R. mucilaginosa* and *R. glutinis* are broadly distributed in our environmental samples and they can also cause diseases in humans [38]. Overall, about 38% of the strains that we isolated from the environmental samples in this study belonged to yeast species that have been reported to cause diseases in humans. The results are consistent with the potential importance of environmental yeasts in human health.

Our study uncovered 144 environment yeast species among 984 isolates, including 38 putative novel species. Among these, *C. tropicalis* was the most common species in both the overall soil and freshwater samples and the third most common in seawater samples. Significantly, 24.6% of the *C. tropicalis* isolates were resistant to fluconazole. Both the *C. tropicalis* isolation rate and drug resistance rate in the environmental samples were higher than those from human oral swab samples in Hainan [12,13]. Similarly, the *C. tropicalis* isolation rate here was also higher than in environmental samples from other countries [2,11]. *C. tropicalis* accounts for a significant proportion of clinical candidiasis worldwide, especially for several geographic areas such as Brazil and southeast Asia. In these regions, *C. tropicalis* is either the first or the second most frequently isolated yeast in humans [39,40,41,42]. The high-frequency isolation of *C. tropicalis* in our current study is consistent with the global pattern for tropical regions. Furthermore, genotype sharing between our samples and those published previously as revealed by MLST is consistent with long-distance gene flow between countries and continents, and between humans and in natural environments [22,42].

Similar to *C. tropicalis* in our environmental samples, the next two highly prevalent environmental yeast species *C. krusei* and *T. delbrueckii* also showed high rates of fluconazole resistance. Specifically, both species are opportunistic human pathogens [3,10], each accounting for 9.2% (90/984) of the total yeast isolates in our samples. Our susceptibility testing revealed that 65.9% of *C. krusei* and 75% of *T. delbrueckii* from Hainan environmental samples were resistant to fluconazole. Previous studies have suggested that the application of agricultural fungicides is a significant driver for the selection of clinical drug-resistant strains of *A. fumigatus* [14,15]. A similar process might be happening in Hainan to drive the evolution of fluconazole resistance in these yeasts. Specifically, the applications of triazole fungicides in orchards, vegetables gardens, and agricultural crop fields in terrestrial environments could be the selection force. Through rainfall and irrigation, some of those drug-resistant strains in terrestrial environments could enter the freshwater system (creeks, rivers, and lakes) and then marine environments. The high frequency of fluconazole resistance of human pathogenic yeasts in environmental samples calls for greater attention to investigate the origins of these fluconazole-resistant environmental strains and to design strategies to reduce their frequencies.

## 5. Conclusions

Yeasts play important roles in the environment and human welfare. In this study, we investigated the diversity of yeasts in tropical environments in China and their potential impacts on human health. Abundant diversity of yeasts was found in the tropical region in China, including many potential new species. The large number of pure yeast cultures obtained here lay solid foundations for future taxonomic, population genetic, genomic, and potential application studies [43]. Furthermore, a significant number of pathogenic yeast species were found in our samples and a significant proportion of these yeasts were tolerant/resistant to the commonly used antifungal drug fluconazole. Our results suggest that these environmental yeasts could have significant negative impacts on human health.

## Figures and Tables

**Table 1:** Summary statistics of yeast isolation from Hainan environment samples.

Sample Types	Sample Characteristics	No. of Samples	No. of Samples with Yeasts (% Oositive/Total)	Yeast Isolates	Known Species	Putative novel Species	Private Species	Shannon Diversity Index
Ecological	Soil	516	369(71.5)	512	88	27	46	3.50
niches	Freshwater	273	234(85.7)	361	52	14	11	2.90
	Seawater along beaches	179	78(43.6)	111	21	2	-	2.41
Geographic	Haikou	120	70(58.3)	97	32	3	9	2.88
regions	Wenchang	131	93(71)	125	27	4	12	2.96
	Lingshui	119	88(74)	114	31	-	7	3.04
	Baoting	135	116(85.9)	191	39	15	9	2.55
	Dongfang	127	95(74.8)	128	20	8	4	2.49
	Danzhou	153	112(73.2)	179	34	6	7	2.66
	Sanya	133	88(66.2)	124	29	2	5	2.22
	Sansha	50	19(38)	26	13	-	4	2.10
Total		968	681(70.4)	984	106	38	57	

**Table 2:** Environmental yeast species with strains having fluconazole intermediate and/or resistance phenotypes as determined by CLSI M44-A2. S: susceptible; I: intermediate; R: resistant.

Species	Total	S	I	R	I+R	(% Total)	Not Tested
*C. tropicalis*	207	150	8	43	51	25.4%	6
*C. krusei*	90	29	32	22	56	65.9%	7
*Torulaspora delbrueckii*	90	21	4	59	63	75.0%	6
*Meyerozyma caribbica*	51	45	0	5	5	10.0%	1
*C. pseudolambica*	48	11	11	24	35	76.1%	2
*Trichosporon asahii*	46	40	0	6	6	13.0%	0
*Diutina rugosa*	40	35	0	3	3	7.9%	2
*Wickerhamomyces sydowiorum*	30	24	0	6	6	20.0%	0
*Rhodotorula toruloides*	20	2	0	17	17	89.5%	1
*Dirkmeia churashimaensis*	18	4	0	9	9	69.2%	5
*C. guilliermondii*	18	12	2	4	6	33.3%	0
*C. quercitrusa*	17	8	5	3	8	50%	1
*C. intermedia*	16	13	0	2	2	13.3%	1
*Kodamaea ohmeri*	16	14	1	1	2	12.5%	0
*C. palmioleophila*	14	1	0	13	13	92.9%	0
*Rhodotorula paludigena*	13	1	0	8	8	88.9%	4
*Diutina mesorugosa*	7	5	0	1	1	16.7%	1
*C. gosingica*	6	4	1	0	1	20%	1
*Cyberlindnera saturnus*	6	1	3	0	3	75.0%	2
*Rhodotorula mucilaginosa*	6	0	0	6	6	100%	0
*Aureobasidium melanogenum*	5	2	1	1	2	50%	1
*C. albicans*	5	4	0	1	1	20%	0
*Diutina catenulata*	5	3	1	1	2	40%	0
*Moesziomyces antarcticus*	5	0	0	2	2	100%	3
*C. dubliniensis*	5	3	0	2	2	40%	0
*C. stellimalicola*	4	2	0	1	1	33.3%	1
*Rhodosporidiobolus ruineniae*	4	1	0	3	3	75%	0
*Rhodotorula glutinis*	4	0	0	1	1	100%	3
*Wickerhamomyces rabaulensis*	4	1	0	3	3	75%	0
*C. nivariensis*	3	2	0	1	1	33.3%	0
*Hanseniaspora opuntiae*	3	2	0	1	1	33.3%	0
*Papiliotrema laurentii*	3	0	1	2	3	100%	0
*Pichia manshurica*	4	0	0	1	3	100%	0
*C. inconspicua*	2	0	1	0	1	100%	1
*Debaryomyces castellii*	2	0	1	1	2	100%	0
*Kwoniella heveanensis*	1	1	0	0	1	50%	0
*Lachancea dasiensis*	2	0	1	0	1	100%	1
*Moesziomyces aphidis*	2	0	0	1	1	100%	1
*Pichia kluyveri*	2	0	0	2	2	100%	0
*Rhodosporidium toruloides*	2	0	0	2	2	100%	0
*Saccharomyces cerevisiae*	2	1	0	1	1	50%	0
*Ustilago sparsa*	2	1	0	1	1	50%	0
*Wickerhamomyces edaphicus*	2	1	0	1	1	50%	0
*Apiotrichum laibachii*	1	0	1	0	1	100%	0
*Apiotrichum mycotoxinovorans*	1	0	0	1	1	100%	0
*C. fennica*	1	0	0	1	1	100%	0
*C. sonorensis*	1	0	0	1	1	100%	0
*Clavispora opuntiae*	1	0	0	1	1	100%	0
*Cryptococcus flavescens*	1	0	1	0	1	100%	0
*Moesziomyces rugulosus*	1	0	0	1	1	100%	0
*Pichia bruneiensis*	1	0	0	1	1	100%	0
*Pichia fermentans*	1	0	0	1	1	100%	0
*Pichia membranifaciens*	1	0	0	1	1	100%	0
*Schwanniomyces vanrijiae*	1	0	1	0	1	100%	0
*Wickerhamiella infanticola*	1	0	0	1	1	100%	0
*Yamadazyma cocois*	1	0	0	1	1	100%	0
*Candida sp.* NEW	14	8	0	6	8	100%	0
*Saturnispora sp.* NEW	1	0	1	0	1	100%	0
*Kalmanozyma sp.* NEW	1	0	0	1	1	100%	0
*Rhodotorula sp.* NEW	15	1	0	11	11	91.7%	2
*Apiotrichum sp.* NEW	1	0	0	1	1	100%	0
*Meyerozyma sp.* NEW	1	0	0	1	1	100%	0

**Table 3:** Comparisons of polymorphisms at the six *C. tropicalis* gene fragments between clinical and environmental samples.

Gene Loci	Clinical Sample (116 Isolates) G/P (Ratio)	Environmental Sample (44 Isolates) G/P (Ratio)	Total (160)
*ICL1*	8/6 (1.3)	6/3 (2.0)	10/6 (1.7)
*MDR1*	37/21 (1.8)	21/12 (1.8)	47/22 (2.1)
*SAPT2*	11/7 (1.6)	8/6 (1.3)	14/10 (1.4)
*SAPT4*	21/18 (1.2)	12/14 (0.9)	28/18 (1.6)
*XYR1*	33/16 (2.4)	22/17 (1.3)	45/18 (2.5)
*ZWFa1*	14/11 (1.3)	9/5 (1.8)	17/11 (1.6)
Total	94/79 (1.2)	33/57 (0.6)	124/85 (1.5)

G: number of genotypes; P: number of polymorphic nucleotide sites; Ratio: ratio of the number of genotypes over the number of polymorphic nucleotide sites.

**Table 4:** Summary table of AMOVA result between clinical and environmental populations of *C. tropicalis*.

Source	df	SS	MS	Est. Var.	%
Among Pops	1	41.609	41.609	0.516	6% ***
Within Pops	158	1369.484	8.668	8.668	94% ***
Total	159	1411.094		9.184	100%

*** *p* = 0.001.

## Data Availability

The data that support the findings of this study are available from the corresponding authors, J.P.X. and J.Y.W., upon reasonable request.

## Supplementary Materials

The following are available online at https://www.mdpi.com/article/10.3390/genes13030444/s1, Table S1: Specific information of sampling and isolated strains, Table S2: Information about strains of *C. tropicalis* from Hainan Island, Table S3: Taxonomy of environment yeast species isolated in this study, Table S4: Information of putative novel species, Table S5: Comparison of six gene fragments in *C. tropicalis* between oral and environmental samples, Table S6: Summary data on susceptibilities to fluconazole among geographic regions and ecological niches.
