# Supplementary material for: Extensive Diversity and Prevalent Fluconazole Resistance among Environmental Yeasts from Tropical China
Source: Genes (Basel). 2022 Feb 28;13(3):444. doi: 10.3390/genes13030444 (PMC8954247; doi:10.3390/genes13030444)
Supplement: Supplementary file 1 [file genes-13-00444-s001.zip › Table S5.pdf]

genes

MDPI

Table S5. Comparison of six gene fragments in *C. tropicalis* between the oral population and the environmental population.

Gene loci	Oral sample (116 isolates)			Environment sample (44 isolates)		
	G /P (Ratio)	New G	Main genotypes	G /P (Ratio)	New G	Main genotypes
ICL1	8/6 (1.33)	1	ICL1-1	6/3(2.00)	0	ICL1-1
MDR1	37/21 (1.76)	14	MDR1-9,22	21/12(1.75)	6	MDR1-16,117
SAPT2	11/7 (1.57)	4	SAPT2-1,3,12	8/6(1.33)	1	SAPT2-3,1
SAPT4	21/18 (1.17)	5	SAPT4-7,17	12/14(0.86)	2	SAPT4-10,3,4,7
XYR1	33/16 (2.36)	8	XYR1-60,9	22/17(1.29)	3	XYR1-76,9
ZWFa1	14/11 (1.27)	5	ZWFa1-22,3	9/5(1.8)	1	ZWFa1-3,1
Total	94/79 (1.19)			32/57(0.58)		

G: the number of genotypes; P: the number of polymorphic nucleotide sites; New G: the number of new genotypes found the first time in the MLST database; Main genotypes: the most frequent genotypes in each sample.
